# EWAS of post-COVID-19 patients shows methylation differences in the immune-response associated gene, *IFI44L*, three months after COVID-19 infection

**DOI:** 10.1038/s41598-022-15467-1

**Published:** 2022-07-07

**Authors:** Yunsung Lee, Espen Riskedal, Karl Trygve Kalleberg, Mette Istre, Andreas Lind, Fridtjof Lund-Johansen, Olaug Reiakvam, Arne V. L. Søraas, Jennifer R. Harris, John Arne Dahl, Cathrine L. Hadley, Astanand Jugessur

**Affiliations:** 1 Centre for Fertility and Health, Norwegian Institute of Public Health, Skøyen, P.O. box 222, 0213 Oslo, Norway; 2 Age Labs AS, Gaustadalléen 23A, 0373 Oslo, Norway; 3 Department of Microbiology, Oslo University Hospital Rikshospitalet, 0372 Oslo, Norway; 4 Department of Microbiology, Oslo University Hospital Ullevaal, 0372 Oslo, Norway; 5 Department of Immunology, Oslo University Hospital Rikshospitalet, 0372 Oslo, Norway; 6 Department of Global Public Health and Primary Care, University of Bergen, P.O. box 7804, 5020 Bergen, Norway

**Keywords:** Epigenetics, Infection

## Abstract

Although substantial progress has been made in managing COVID-19, it is still difficult to predict a patient’s prognosis. We explored the epigenetic signatures of COVID-19 in peripheral blood using data from an ongoing prospective observational study of COVID-19 called the Norwegian Corona Cohort Study. A series of EWASs were performed to compare the DNA methylation profiles between COVID-19 cases and controls three months post-infection. We also investigated differences associated with severity and long-COVID. Three CpGs—cg22399236, cg03607951, and cg09829636—were significantly hypomethylated (FDR < 0.05) in COVID-19 positive individuals. cg03607951 is located in *IFI44L* which is involved in innate response to viral infection and several systemic autoimmune diseases. cg09829636 is located in *ANKRD9*, a gene implicated in a wide variety of cellular processes, including the degradation of IMPDH2. The link between *ANKRD9* and *IMPDH2* is striking given that IMPDHs are considered therapeutic targets for COVID-19. Furthermore, gene ontology analyses revealed pathways involved in response to viruses. The lack of significant differences associated with severity and long-COVID may be real or reflect limitations in sample size. Our findings support the involvement of interferon responsive genes in the pathophysiology of COVID-19 and indicate a possible link to systemic autoimmune diseases.

## Introduction

Coronavirus disease 2019 (COVID-19) is caused by the highly contagious severe acute respiratory syndrome coronavirus 2 (SARS-CoV-2) and is marked by a wide spectrum of symptoms. Although most patients with COVID-19 experience a mild disease course, up to 35% report long-term sequelae, including memory problems, depression, dyspnea, anosmia, ageusia, and fatigue lasting months after the initial infection^1,2^. These are among the symptoms that characterize a syndrome referred to as Post-Acute Sequelae of SARS-CoV-2 infection (PASC) or “long-COVID”. The causes and persistence of long-COVID are not well understood, although several hypotheses have been proposed, including damage to the autonomic nerve system caused by inflammation^3^, the formation of autoantibodies in susceptible individuals^4^, and viral persistence. The lack of detectable antibodies in half of the patients presenting symptoms attributed to SARS-CoV-2 in another study^5^ points to a possible confounding etiology such as pandemic stress.

Evidence points to subtle differences in the immunological profiles of individuals with long-COVID compared to those who recover quickly. The syndrome seems to occur regardless of the development of long-term immunity, as antibody levels have been found to be similar four months after COVID-19 symptom onset to that of convalescents^6^. However, participants with long-COVID appear to have a rapidly waning number of interferon gamma (IFN-γ) producing CD8+ cells four months after COVID-19 symptom onset^6^. This is interesting, as cytotoxic lymphocytes, particularly CD8+ lymphocytes and Natural Killer (NK) cells, affect viral clearance directly or through the release of IFN-γ^7^. An improved understanding of the role of host factors in COVID-19 is necessary to improve the diagnosis of long-COVID and develop efficient treatments.

DNA methylation (DNAm) is a stable epigenetic mark that regulates gene expression without altering the DNA sequence itself. It plays a pivotal role in normal biological processes as well as in the pathogenesis of diseases^8–10^. An epigenome-wide association study (EWAS) entails studying DNAm differences associated with a phenotype at hundreds of thousands of cytosine-phosphate-guanine (CpG) sites across the epigenome. The human epigenome harbors an estimated 28 million CpG sites^11^, of which 60–70% are located near transcription start sites^12^. Differentially methylated regions (DMRs) represent genomic regions with different DNAm levels between a case and a control group. The effect of methylation of a given CpG site depends on the genomic context^13^. For instance, hypermethylation of CpG sites in a gene body is commonly associated with higher gene expression^14^.

Genomic studies have revealed key susceptibility genes for COVID-19^15–18^, the most prominent of which is angiotensin-converting enzyme 2 (*ACE2*)^19^. Epigenetic investigations add another layer of omics analysis to the study of SARS-CoV-2 and reveal that methylation patterns are predictive of disease severity^20–22^. Three major considerations in deciphering the link between epigenetic variation and infection are how the host responds to viral exposure, how the virus exploits the host’s epigenome to establish infection, and how innate susceptibility differs between individuals. It remains unknown whether the DNAm differences reported thus far in COVID-19 reflect differences in susceptibility, temporary immune modulation, or more permanent changes.

To date, none of the published studies on long-COVID have investigated DNA methylation. Our aim was therefore to explore epigenetic signatures that are correlated with the severity of COVID-19 and the onset of long-COVID. To this end, we conducted a series of EWASs where we compared DNAm profiles in the peripheral blood of 109 Norwegians, eight to twelve weeks after SARS-CoV-2 infection, with the corresponding profiles of 73 controls.

## Results

### Characteristics of the sample population

**Figure 1:**
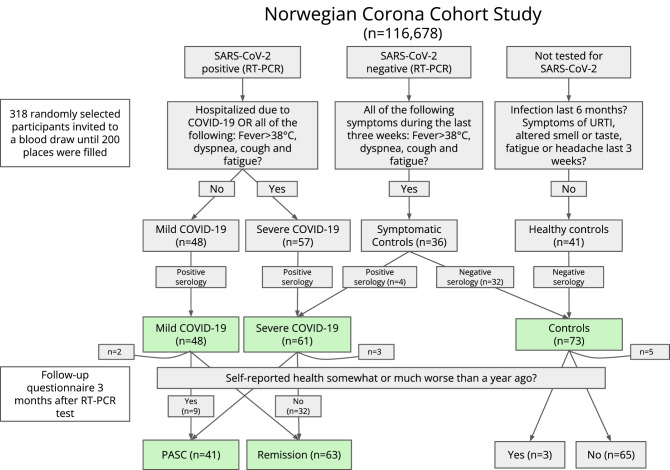
Overview of the sampling scheme. 184 of the 200 participants who agreed to a blood draw showed up for their appointment and completed the baseline questionnaire. Two participants lacked data and were excluded from the study, bringing the final number of participants to 182. Four participants with a negative SARS-CoV-2 RT-PCR test and who reported symptoms of severe COVID-19 had a positive serology test. They were considered false negatives and reclassified as severe COVID-19. Ten participants were treated as ‘lost to follow-up’, as they did not return the three-month follow-up questionnaire despite reminders. The control group was not included in the long-COVID EWAS analysis.

Figure 1 and Table 1 provide details of the sample selection and the study population according to COVID-19 status, sex, mean age, smoking history, presence of chronic diseases, and the average number of days from the time the RT-PCR test was performed to (i) the time of enrolment (hereafter referred to as ‘baseline’), (ii) the blood draw, and (iii) the follow-up questionnaire. Note that the healthy controls did not undergo an RT-PCR test. Among the symptomatic controls with a negative RT-PCR test, four had a positive serology test and were therefore considered false negatives. As these four cases had reported both fever and dyspnea, they were reclassified as belonging to the severe COVID-19 category. The remaining 32 symptomatic controls with a negative RT-PCR test had a confirmatory negative serology test and were thus grouped together with the 41 healthy controls in the control category (n = 73).

**Table 1:** Characteristics of the study population according to COVID-19 status.

Characteristics	COVID-19 (n = 109)		Controls (n = 73)
	Severe COVID-19^†^ (n = 61)	Mild COVID-19 (n = 48)	
Female	39 (64)	27 (56)	50 (68)
Age median (IQR)	45 (34–54)	55 (45–65)	49 (38–55)
Chronic disease	22 (36)	14 (29)	28 (38)
Smoking history	22 (36)	19 (40)	31 (42)
Asymptomatic	0 (0)	5 (10)	41 (56)
Long-COVID^‡^	32 (52)	9 (19)	NA NA
**Self-reported health compared to one year ago**			
Much worse now than one year ago	7 (11)	0 (0)	0 (0)
Somewhat worse now than one year ago	25 (41)	9 (19)	3 (4)
About the same	20 (33)	35 (73)	52 (71)
Somewhat better now than one year ago	6 (10)	2 (4)	9 (12)
Much better now than one year ago	0 (0)	0 (0)	4 (5)
Missing	3 (5)	2 (4)	5 (7)
Self-reported dyspnea at follow-up	14 (23)	2 (4)	2 (3)
Self-reported fatigue at follow-up	19 (31)	10 (21)	12 (16)
Self-reported change in smell or taste at follow-up	10 (16)	3 (6)	0 (0)
Median days from RT-PCR to baseline (IQR)	9 (5–20)	15 (10–19)	12* (6–18)
Median days from RT-PCR to blood draw (IQR)	67 (62–75)	67 (63–76)	70* (68–71)
Median days from RT-PCR to follow-up questionnaire (IQR)	105 (105–120)	111 (101–120)	109* (108–117)

Numbers enclosed in parentheses indicate the percentages, except when referring to the interquartile range (IQR).

*NA* Not applicable.

^†^Of the severe cases, seven were hospitalized.

^‡^Those reporting worse health compared to one year ago at follow-up were defined as PASC, while the remainder was defined as the remission group.

*The control group consisted of 41 healthy controls and 32 symptomatic controls with other upper respiratory tract infection (URTI). Note that the 41 healthy controls did not undergo any RT-PCR testing. The three participants in the control group who reported dyspnea, fatigue and worse self-reported health compared to one year ago at the three-month follow-up were all among the symptomatic controls.

36% to 42% of the participants reported smoking in each of the three main categories. Additionally, 15% of the mild COVID-19 cases had at least one chronic disease; the corresponding proportion was 36% among the severe COVID-19 cases and 38% among the controls. At three months of follow-up, 52% of the severe COVID-19 cases reported that their health was worse than a year ago; the corresponding proportion was 19% among the mild cases, 9% among the symptomatic controls, and none among the healthy controls. Among the severe cases, 31% reported fatigue and 23% dyspnea three months after the infection.

### Comparison of DNAm profiles

**Figure 2:**
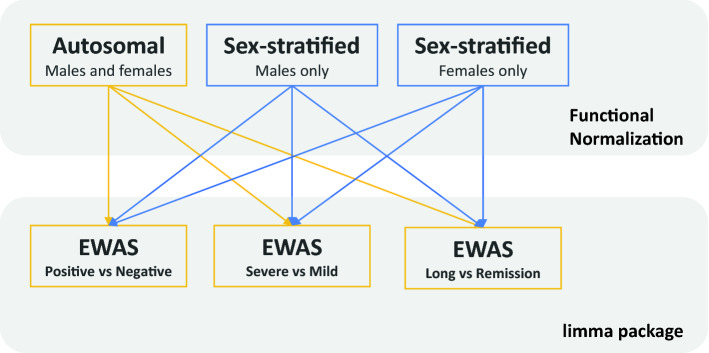
Overview of the analytic pipeline. An EWAS was performed for each of the following comparisons: (i) COVID-19 positive *vs* COVID-19 negative, (ii) severe COVID-19 *vs* mild COVID-19, and (iii) long-COVID *vs* the remission group (labeled ‘Remission’ in the figure). Additional comparisons are provided in Supplementary Figs. 8 and 9. We applied the R package limma^63^ on functional normalized M-values in each EWAS. In total, we conducted nine separate EWASs.

**Figure 3:**
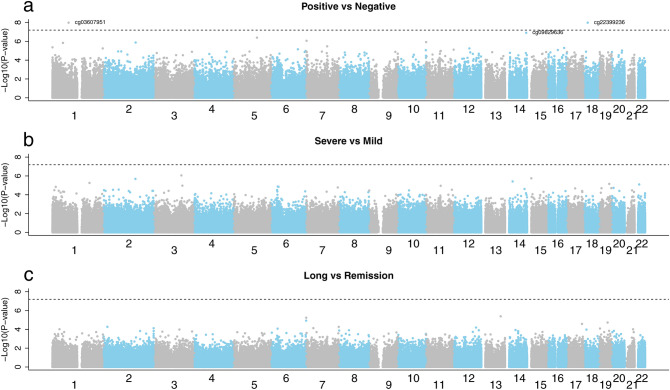
Manhattan plots for the EWASs. The Manhattan plots display the association statistics at each of the 776,892 autosomal probes between (**a**) COVID-19 positive (n = 109) and COVID-19 negative (n = 73), (**b**) severe COVID-19 (n = 61) and mild COVID-19 (n = 48), and (**c**) long-COVID (n = 41) and remission (n = 63). The dotted horizontal line is the Bonferroni threshold (0.05/776,892 CpG sites), and the labeled dots are the significant CpGs at FDR < 0.05.

**Figure 4:**
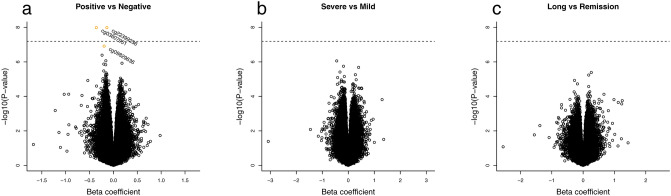
Volcano plots for the pooled sample of males and females in the analysis of autosomal probes. The plots display the estimated beta coefficients against −log_10_ of the P-values. (**a**) COVID-19 positive (n = 109) versus COVID-19 negative (n = 73), (**b**) severe COVID-19 (n = 61) versus mild COVID-19 (n = 48), and (**c**) long-COVID (n = 41) versus remission (n = 63). The dotted horizontal line refers to the Bonferroni threshold (0.05/776,892 CpG sites), and the orange-colored dots are the significant CpGs at FDR < 0.05.

**Figure 5:**
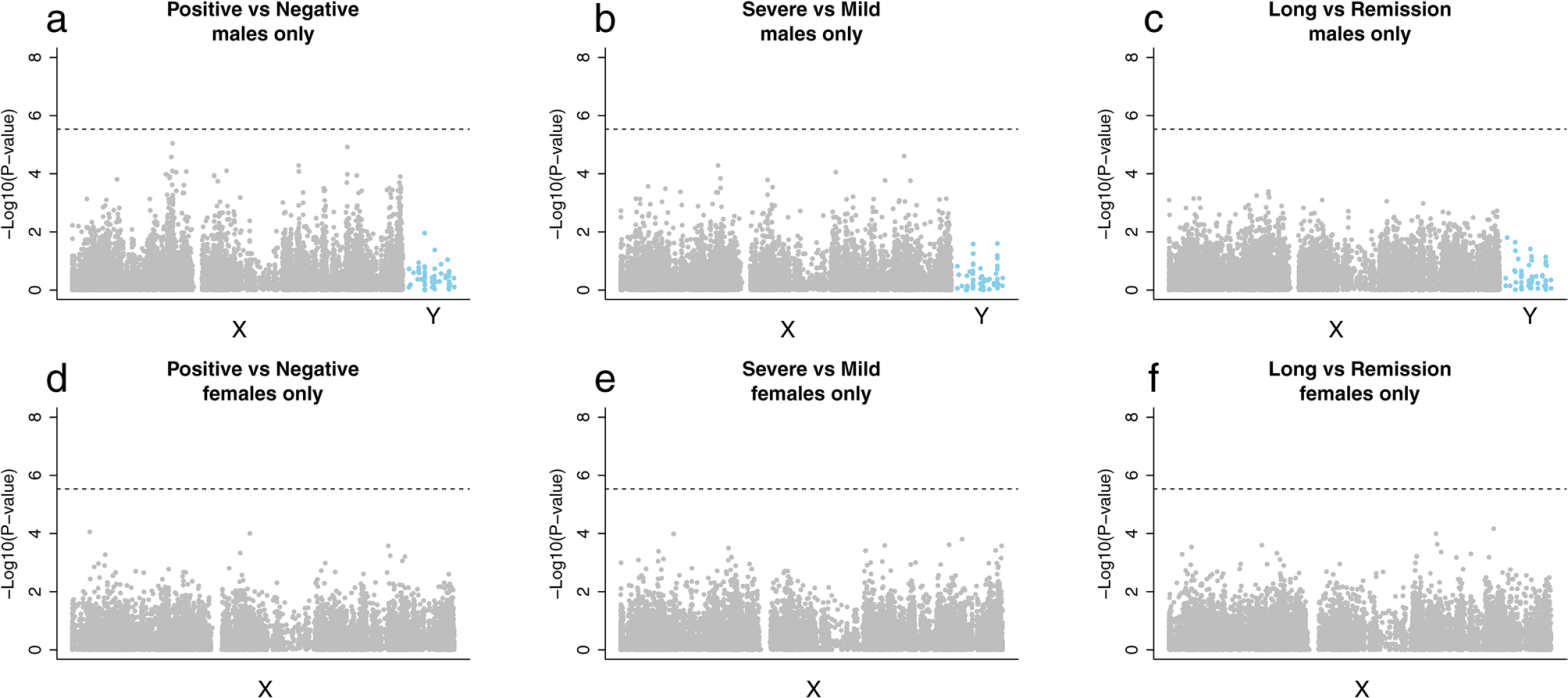
Manhattan plots for the sex-stratified EWASs of probes on the sex chromosomes. The plots display the association statistics at each of the 17,183 X-chromosome probes and 51 Y-chromosome probes (indicated by blue dots). The upper panels (**a**) to (**c**) are for males only (including the Y-chromosome probes), and the lower panels (**d**) to (**f**) are for females only (X-chromosome probes only). Panels (**a**) and (**d**) display the result of the comparison between COVID-19 positive and COVID-19 negative. Panels (**b**) and (**e**) show the results of the comparison between severe COVID-19 and mild COVID-19. Panels (**c**) and (**f**) show the results of the comparison between long-COVID and remission. The dotted line indicates the Bonferroni threshold (0.05/17,183 CpG sites).

**Figure 6:**
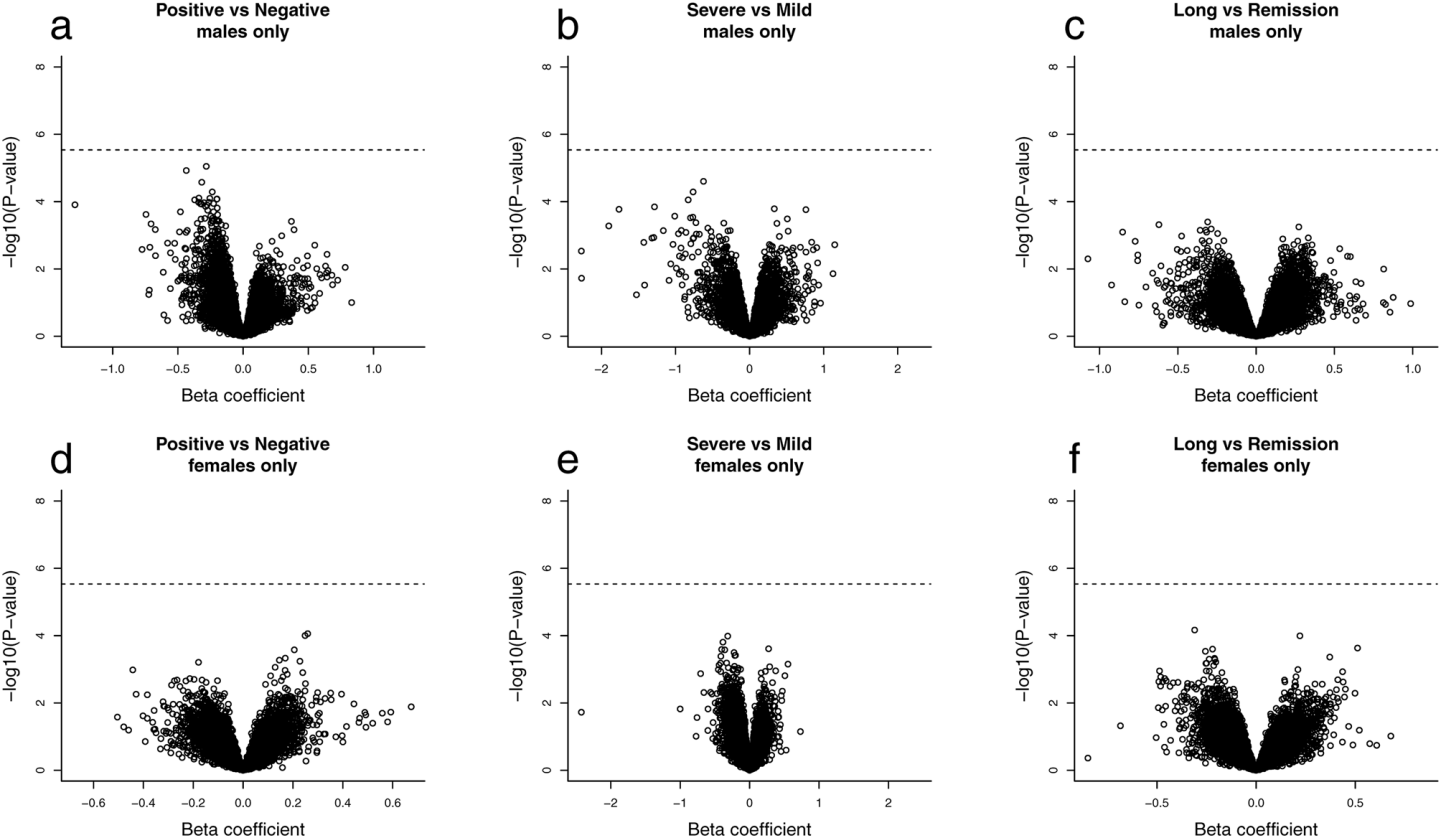
Volcano plots for the sex-stratified analyses targeting sex-chromosome probes. The plots display the estimated beta coefficients against -log_10_ of the P-values. Panels (**a**) to (**c**) are for males only and panels (**d**) to (**f**) are for females only. Panels (**a**) and (**d**) show the results of the COVID-19 positive (n = 109) versus COVID-19 negative (n = 73) comparison. Panels (**b**) and (**e**) show the results of the severe COVID-19 (n = 61) versus mild COVID-19 (n = 48) comparison. Panels (**c**) and (**f**) show the results of the long-COVID (n = 41) versus remission (n = 63) comparison. The dotted horizontal line indicates the Bonferroni threshold (0.05/17,183 CpG sites for males and 0.05/17,128 CpG sites for females).

We adopted the analytic strategy outlined in Fig. 2. Specifically, data from males and females were analyzed together for autosomal probes (Figs. 3 and 4, and Supplementary Fig. 1), and separately for sex-chromosome probes (Figs. 5 and 6, and Supplementary Fig. 2). The sex-stratified analyses were motivated by the presence of distinct sex differences in the distribution of mean methylation values on the sex chromosomes (Supplementary Fig. 3). Our primary objective was to compare the DNAm profiles of the following categories of study participants: 1) COVID-19 positive (n = 109) versus COVID-19 negative (n = 73), 2) severe COVID-19 (n = 61) versus mild COVID-19 (n = 48), and 3) long-COVID (n = 41) versus remission (n = 63) (see Fig. 2 and “Methods” for details). We adjusted for the following variables in each EWAS: age, sex, smoking pack-years (estimated from the DNAm data), physical fitness, and estimates of white blood cell counts, i.e., CD8+ naïve and exhausted cytotoxic T cells, CD4+ naïve cells, NK cells, B cells, monocytes, and granulocytes.

The severe COVID-19 group differed significantly from the other groups in terms of cell-type composition. Notably, the B-lymphocyte proportion was greater in the severe COVID-19 group than in the other groups. Additionally, this group differed with regards to the composition of CD8+ T cells, granulocytes, and NK cells (Supplementary Figs. 4 and 5). There were no significant differences in cell type composition between the long-COVID group and the remission group. A comparison of epigenetic age acceleration (EAA) between the groups is presented in Supplementary Fig. 6. We did not find any significant differences in EAA between the groups when we used the DNAmAge clock.

### Pooled analyses (males and females combined)

The methylation levels at three CpGs differed significantly (false discovery rate (FDR) < 0.05) in the comparison between COVID-19 positive (n = 109) and COVID-19 negative (n = 73) (Fig. 3). All the CpGs were hypomethylated. The first CpG, cg22399236, is located on chromosome 18 and is not close to any known gene within at least 30 kb, according to a search in the UCSC Genome Browser using GRCh37/hg19. The second CpG, cg03607951, is in the gene ‘interferon induced protein 44 like’ (*IFI44L*) on chromosome 1p31.1. Groupwise comparisons of differences in the methylation of this CpG is presented in Supplementary Fig. 7. The third CpG, cg09829636, is in the gene ‘ankyrin repeat domain 9’ (*ANKRD9*) on chromosome 14q32.31. Among the 109 individuals with information on severity of COVID-19 symptoms, we compared the DNAm levels between 61 cases with severe symptoms and 48 cases with mild symptoms. No statistically significant methylation differences were detected. Further, we compared the DNAm levels between 41 cases classified as having long-COVID and 63 cases in remission. No statistically significant methylation differences were detected.

**Figure 7:**
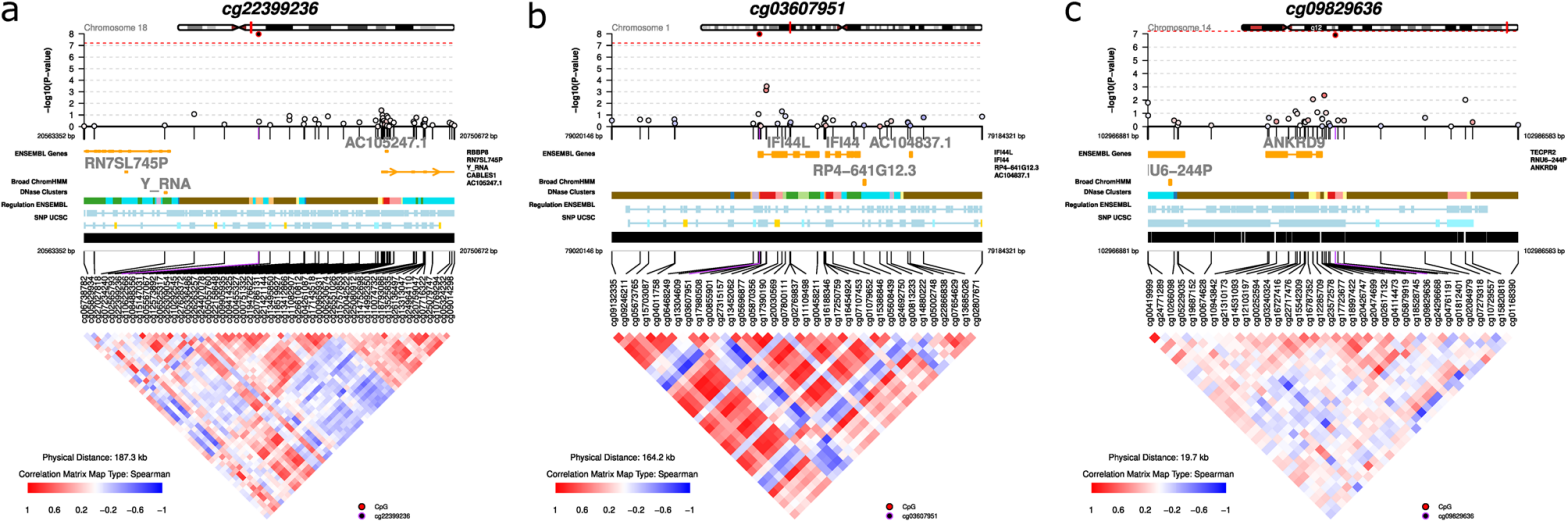
Visualization of the three differentially methylated CpGs identified in the COVID-19 positive versus COVID-19 negative comparison. (**a**) cg22399236 (chr18:20651637) (**b**) cg03607951 (chr1:79085586) in *IFI44L* (**c**) cg09829636 (chr14:102976856) in *ANKRD9*. The correlation map underneath each plot represents pairwise correlations between any two probes (red for high correlation and blue for low correlation). The plot was generated using the coMET R package^71^.

To visualize the local correlation structure of the three significant CpGs, we generated regional co-methylation plots for each CpG using the coMET R package. cg03607951 and cg09829636 showed a moderate degree of correlation with neighboring CpGs (red patches in the heat map in Fig. 7), whereas cg22399236 showed weak correlations.

In addition, we queried the online mQTL database^23^ to investigate whether methylation of these CpGs is influenced by common SNPs; in other words, we searched for methylation quantitative trait loci (mQTLs) associated with these CpGs. Both cg03607951 and cg09829636 were associated with several trans-acting mQTLs, but the search output did not show any entries for cg22399236 (Supplementary Table 1).

### Sex-stratified analyses

As males and females show distinctively different mean methylation values on the sex chromosomes (Supplementary Fig. 3), we performed sex-stratified analyses for probes on the sex chromosomes. No significant differences (FDR < 0.05) in methylation values of CpGs were detected in any of the analyses. However, it may be worth noting that the methylation levels at two CpGs on the X chromosome were slightly lower in the COVID-19 positive males than the COVID-19 negative males (Fig. 6). These were cg08118341 (P = 8.96e−06, chrX:48931823) near the gene ‘PRA1 domain family member 2’ (*PRAF2*), and cg24340926 (P = 1.19e−05, chrX:129305036) near the gene ‘RAB33A, member RAS oncogene family’ (*RAB33A*).

### Location of significant CpGs

We searched for the location of the three significant CpGs using the *Ensembl* browser^24^. cg03607951 is located on chromosome 1 (Chr1:79085586–79085635) and cg09829636 on chromosome 14 (Chr14:102976856–102976905). cg03607951 and cg09829636 are both located in gene promoter regions (of *IFI44F* and *ANKRD9*, respectively), which is not surprising given that most of the CpGs on the Illumina 450 K array that were migrated over to the more recent Illumina EPIC array are predominantly located within gene promoters and promoter-flanking regions. *Ensembl* did not return any output for cg22399236, which is probably because this CpG was not present on the former Illumina 450 K platform on which the *Ensembl* entries are based. The Infinium MethylationEPIC Manifest File^25^ indicates that this CpG is located on chromosome 18 (nucleotide position 20651395 based on GRch37) and corresponds to the SNP rs576870425. According to the ‘Functional annotation of the mammalian genome 5’ (FANTOM5) database^26^ and the Illumina Manifest File for the EPIC array, this CpG overlaps with one regulatory feature, ENSR00001016303, which is an enhancer region. Moreover, the *Ensembl* Regulatory Build indicates that this CpG is located within a promoter-flanking region. Thus, even though cg22399236 is located in a ‘gene desert’ on chromosome 18, it may have regulatory (enhancer) functions in genes located nearby.

### Differentially methylated regions

We identified 168 differentially methylated regions (DMRs) from the nine sets of comparisons (Supplementary File 3). An example of a significant DMR with a low P-value was the genomic region chr6:33245490–33246043, containing 19 CpG sites and located near the gene ‘beta-1,3-galactosyltransferase 4’ (*B3GALT4*). This DMR was less methylated in the COVID-19 positive group (males and females combined) than the COVID-19 negative group (P = 1.24e−15, Bonferroni-corrected P = 1.04e−09). Another example of a significant DMR was the region on chr3:21792248–21792991, containing nine CpGs and located near ‘Zinc Finger Protein 385D’ (*ZNF385D*). This DMR was differentially methylated in the severe COVID-19 group compared to the mild COVID-19 group (P = 4.82e−14, Bonferroni-corrected P = 4.00e−14). Lastly, two highly significant DMRs were detected in the long-COVID versus remission comparison (chr6:32121225–32121555 near ‘palmitoyl-protein thioesterase 2’ (*PPT2*) and chr14:63671314–63671737 near ‘ras homolog family member J’ (*RHOJ*))*.*

### Gene-set enrichment analysis

Table 2 shows the summary statistics for the top 20 gene ontology (GO) enrichment results. Although we did not detect any statistically significant (FDR < 0.05) gene-set enrichment in the GO or “Kyoto Encyclopedia of Genes and Genomes” (KEGG) pathways, the results of the top 20 GO pathways were enriched for several terms related to defense against viral infection. These enrichments were all related to cg03607951 in *IFI44L*. By contrast, the results of the KEGG analysis did not identify any pathways.

**Table 2:** The top 20 most enriched gene ontology (GO) pathways for the comparison between COVID-19 positive and COVID-19 negative individuals.

GO ID	ONTOLOGY	TERM	N	DE	P.DE	FDR
GO:0051607	BP	Defense response to virus	254	1	0.024	1
GO:0009615	BP	Response to virus	344	1	0.033	1
GO:0005525	MF	GTP binding	366	1	0.039	1
GO:0043687	BP	Post-translational protein modification	360	1	0.039	1
GO:0032550	MF	Purine ribonucleoside binding	370	1	0.040	1
GO:0032549	MF	Ribonucleoside binding	373	1	0.040	1
GO:0001883	MF	Purine nucleoside binding	373	1	0.040	1
GO:0001882	MF	Nucleoside binding	379	1	0.041	1
GO:0019001	MF	Guanyl nucleotide binding	387	1	0.041	1
GO:0032561	MF	Guanyl ribonucleotide binding	387	1	0.041	1
GO:0098542	BP	Defense response to other organisms	1080	1	0.103	1
GO:0002252	BP	Immune effector process	1180	1	0.114	1
GO:0051707	BP	Response to other organisms	1432	1	0.134	1
GO:0043207	BP	Response to external biotic stimulus	1434	1	0.134	1
GO:0009607	BP	Response to biotic stimulus	1466	1	0.137	1
GO:0006952	BP	Defense response	1685	1	0.152	1
GO:0035639	MF	Purine ribonucleoside triphosphate binding	1789	1	0.158	1
GO:0032555	MF	Purine ribonucleotide binding	1854	1	0.164	1
GO:0032553	MF	Ribonucleotide binding	1871	1	0.165	1
GO:0017076	MF	Purine nucleotide binding	1868	1	0.165	1

‘BP’ stands for biological process, ‘MF’ for molecular function, ‘N’ for number of genes in the GO term, ‘DE’ for number of genes found to be differentially methylated, ‘P.DE’ for P-value for over-representation of the GO term, and ‘FDR’ for false discovery rate.

## Discussion

The primary objective of this study was to determine whether the DNAm profiles of different groups of COVID-19 patients (severe and mild) differed from those of control individuals eight to twelve weeks after infection with SARS-CoV-2. A secondary objective was to investigate whether the DNAm profiles of individuals with long-COVID differed from those in remission. Overall, only the comparison between the COVID-19 positive and COVID-19 negative individuals revealed differentially methylated CpGs at FDR < 0.05 (specifically, cg22399236, cg03607951, and cg09829636). The analyses of COVID-19 severity and long-COVID did not identify any CpGs with significantly different methylation levels.

The comparison between the COVID-19 positive and COVID-19 negative individuals revealed three differentially methylated CpGs at FDR < 0.05 (cg22399236, cg03607951, and cg09829636). A search of these CpGs in the EWAS atlas^27^ showed multiple entries linking hypomethylation of cg03607951 in *IFI44L* to systemic lupus erythematosus (SLE)^28^, primary Sjögren’s syndrome^29^, mixed connective tissue disease^30^, and multiple other autoimmune disorders^31^. By contrast, there were no entries for cg22399236 and cg09829636 in the EWAS atlas. Further, a search in the online mQTL database showed several *trans* mQTLs associated with cg03607951 and cg09829636, but no *cis* or *trans* mQTLs associated with cg22399236. *Trans* mQTLs are known to be more polygenic than *cis* mQTLs and to explain less of the trait variance than *cis* mQTLs^32^. More studies are needed to elucidate how these *trans* mQTLs affect methylation levels at the two CpGs in relation to COVID-19.

*IFI44L* plays an important role in interferon-induced innate viral response and protection against disease. It has targeted antiviral specificity to several viral species^33^. Such a function is not surprising given that *IFI44L* is a paralog of *IFI44*, a key gene involved in the induction of type I and type III interferon signaling^34,35^. However, a recent study showed that inhibition of *IFI44L* impairs the replication of several viruses, while the expression of *IFI44L* impairs antiviral response^34^. Based on these findings, it is interesting to note that a recent transcriptomics study concluded that asymptomatic SARS-CoV-2 infection might be due to decreased expression of six genes, one of which was *IFI44L*^36^. Another transcriptomics study of myocardial tissue from SARS-CoV-2-positive autopsy cases revealed an upregulation of two interferon-related genes (*IFIT3* and *IFI44L*) among those with cardiac involvement^37^. Transcriptome data from human bronchial epithelial cells during SARS-CoV-2 infection have also identified *IFI44L* as one of the top genes upregulated in severe COVID-19^38,39^.

Collectively, the above studies indicate that downregulation of *IFI44L* early in the disease course may be a beneficial physiologic response to control SARS-CoV-2 infection and that it may be associated with a positive outcome. We did not find any methylation differences between severe and mild COVID-19 three months after the infection. However, this may be because both the mild and severe group in our study consisted of outpatients who all survived. Although nonsignificant, the severe COVID-19 group in our study did show a higher expression of *IFI44L*.

The observed hypomethylation of *IFI44L* three months after infection in our study could reflect that it takes time to reverse the immune responses induced by infection. However, it could also reflect a physiological response to control the infection, as complete viral clearance may take months to achieve in some individuals^40,41^. Another explanation is that it could indicate a possible link between COVID-19 and systemic autoimmune diseases. Notably, Zhao et al*.*^42^ proposed that hypomethylation of the *IFI44L* promoter might be a reliable biomarker for the diagnosis of SLE. Furthermore, SARS-CoV-2 has been shown to induce autoantibodies (e.g., antiphospholipid antibodies and transient lupus anticoagulant^43,44^) and trigger autoimmune responses such as hemolytic anemia, thrombocytopenia, and myocarditis^44^. There are recent reports of SARS-CoV-2 infection preceding various autoimmune diseases, including SLE^45,46^. Therefore, longitudinal data allowing an evaluation of the duration of the methylation changes post infection might enhance our understanding of possible long-term effects.

Besides cg03607951 in *IFI44L*, the comparison between COVID-19 positive and COVID-19 negative individuals identified a statistically significant CpG (cg09829636) in ‘ankyrin repeat domain 9’ (*ANKRD9*). ANKRD9 is a highly conserved protein that modulates the intracellular properties of the cytosolic enzyme inosine monophosphate dehydrogenase 2 (IMPDH2) and facilitates its degradation^47^. The link between *ANKRD9* and *IMPDH2* is compelling in light of the role of IMPDHs as therapeutic targets for COVID-19. Specifically, the IMPDH inhibitor, Ribavirin (*aka* tribavirin), is an antiviral medication used for the treatment of COVID-19^48^. Thus, hypomethylation of *ANKRD9* in COVID-19 patients might reflect a physiologic response to control the infection.

The top GO pathways identified in our analyses, although nonsignificant at FDR < 0.05, suggest that *IFI44L* has a central role in viral response. Indeed, cg03607951 in *IFI44L* seemed to be the sole contributor of the three significant CpGs in the GO pathways related to viral response. Our top GO pathway (GO:0051607) “Defense response to virus” has also been reported in two other transcriptomic studies of COVID-19-infected humans and mammals^49,50^.

Our analysis comparing the long-COVID group to the remission group did not identify any significant methylation differences. The lack of significant findings may be real or reflect the small sample size available for this comparison. The proportion of patients evaluated to have long-term symptoms was similar to the numbers reported in previous studies (38% *vs* 33% and 40%)^1,2,51^.

To our knowledge, the current study is the only EWAS of post-COVID-19 and long-COVID patients. Other EWASs of COVID-19 in the literature include those by Castro de Moura et al*.*^20^, Balnis et al*.*^22^, Zhou et al*.*^52^ and a multi-omics study by Bernardes et al*.*^53^. These studies examined DNAm changes early in the disease course, i.e., during the ongoing infection when the immune system is highly activated and found distinct patterns correlating with disease severity. Castro de Moura et al*.* included data on mild and severe COVID-19 cases without underlying conditions, and their DNAm data were also generated on the Illumina EPIC platform. Consistent with our findings, Castro de Moura et al. also found hypomethylation of *IFI44L* among their top ten genes associated with severe COVID-19 early in the disease course. They concluded that the methylation changes were likely due to innate susceptibility rather than changes induced by the virus itself. Apart from *IFI44L*, we had no other overlapping findings with the study by Castro de Moura et al. This may be due to differences in study populations (we only had seven patients in need of ventilator support). It could also be that the methylation signals at the other loci were transient and were reversed three months after infection.

Balnis et al*.*^22^ also had data on COVID-19 positive and COVID-19 negative individuals, and like the above-mentioned study by Castro de Moura et al*.*^20^, their DNAm data were also generated on the Illumina EPIC platform. Despite these similarities, Balnis et al. did not detect any global mean methylation differences between the two groups of participants. Again, this could be due to differences in the study populations (the controls in Balnis et al*.* were an intensive care population). The authors did, however, detect DMRs in another interferon-induced gene, ‘interferon alpha inducible protein 27’ (*IFI27*), as well as in ‘2′-5′-oligoadenylate synthetase 2’ (*OAS2*) which is a member of the 2–5 A synthetase family known to be involved in the innate immune response to viral infection*.* The results of their gene ontology and pathway enrichment analysis were also congruent with ours, pointing to host-defense responses and terms such as ‘response to type I interferon’ and ‘response to virus’, among others.

Despite limited sample sizes (n = 21 and n = 13), the studies by Zhou et al*.*^52^ and Bernardes et al*.*^53^ reported significant methylation changes between mild and severe COVID-19. Zhou et al*.* reported the downregulation of four genes, in particular “G Protein Subunit Gamma 7” (*GNG7*) and “Guanine nucleotide binding protein” (*GNAS*), among patients with severe disease. Likewise, the longitudinally combined transcriptomic and methylation analysis by Bernardes et al*.* revealed the downregulation of *GNG7* and *GNAS2* in severe COVID-19. However, neither study detected methylation changes related to *IFI44L* or *ANKRD9.* The epigenome-wide investigations described above collectively point to innate response to viral infection via interferon-inducible proteins as a possible mechanism for COVID-19 disease progression. In support, our gene-enrichment pathway analysis revealed enrichment of GO terms that were specific for defense responses to viral infection.

Although we did not collect peripheral blood mononuclear cells (PBMCs), Su et al*.* did not find any link to *IFI44L* when examining RNA expression in samples taken three months after SARS-CoV-2 infection in subjects with long-COVID^54^.

Although there have been reports of EAA in severe COVID-19^21^, we did not find any differences in EAA in our study. Interestingly, Cao et al.^55^ found a dynamic increasing EAA in the initial phases of COVID-19, while this increase was partly reversed in the convalescent phase, indicating that the infection might accelerate epigenetic aging. Another study by Mongelli et al*.*^56^ reported increased EAA in younger but not older COVID-19 survivors. More studies are needed to determine the association between EAA and SARS-CoV-2 infection.

### Strengths and limitations of the study

Only a few studies have reported blood-based DNAm in COVID-19, and, to our knowledge, there are no prior reports comparing differentially methylated CpGs three months after SARS-CoV-2 infection. Our study also includes data from confirmatory serology tests performed on all participants, including those with long-COVID, which helps to minimize false positives/negatives. Our study is also based on prospective follow-up questionnaires with high response rates, enabling detailed longitudinal assessments.

Our study also has a few limitations. The evaluation of disease severity was based on self-reported symptoms, and not on an objective assessment. It is difficult to gauge the extent to which recall bias and other types of misclassifications might have influenced the results presented here. The evaluation of long-COVID using the RAND 36-item health survey questionnaire is known to have high sensitivity; however, specificity may be low. Notably, 12% of the Norwegian population reported that their health had, in general, worsened compared to the preceding year. At the same time, the population included in the current study was apparently healthier than the general population. For instance, in the first wave of the pandemic, the COVID-19 positive individuals reported higher physical fitness and socioeconomic status, which is also reflected in our control group where only 4% of the participants reported worse health compared to a year before. Additionally, the evaluation of long-COVID versus remission was likely affected by the small sample size.

In addition, we were unable to explore the expression of *IFI44L* and *ANRKD9* in PBMCs, as extracting these cells from whole blood after freezing is not feasible. The study setup we had at the time of recruitment did not allow for repeated measurements on the same patients both in the acute phase of infection and after three months, which would have provided relevant longitudinal information rather than a single “snapshot” of disease progression.

In conclusion, our study adds to the growing knowledgebase regarding epigenetic contributions to COVID-19. It confirmed previously reported associations with *IFI44L* and the involvement of interferon-responsive genes in the underlying pathophysiology of COVID-19 and showed that such signals can be identified months after the infection. We identified a novel link to *ANKRD9*, which is noteworthy given that IMPDH inhibitors are used in the treatment of COVID-19. This and the other genes identified here would need to be replicated in other EWASs before being dismissed as false positives. We have thus provided all the results of our main EWASs, so that other researchers would be able to easily compare their results to ours.

## Methods

### Study design

The study was designed as a retrospective case–control study nested within the Norwegian Corona Cohort Study (ClinicalTrials.gov Identifier: NCT04320732), which is an ongoing prospective observational study established in March 2020 during the first wave of the COVID-19 pandemic. The cohort consists of two subgroups: (i) adults with a conclusive SARS-CoV-2 RT-PCR test (n = 23,948) invited through four laboratories in the greater Oslo area (Oslo University Hospital, Akershus University Hospital, Vestre Viken hospital, and Fürst Medical Laboratory), and (ii) adults signing up to the study through a media campaign (n = 92,730). All the participants completed an online baseline questionnaire upon enrollment and were invited to follow-up questionnaires at three and six months into the study. Whole blood and serum samples from 110 confirmed COVID-19 cases and 74 controls were retrospectively collected on the 27th and 28th of May 2020 at the Oslo University Hospital.

### Inclusion/exclusion criteria

Participants who were enrolled in the Norwegian Corona Cohort Study and who lived in the greater Oslo area were invited to participate in the current study. They were categorized into three groups based on the following criteria:

- *Severe COVID-19* Participants with a positive SARS-CoV-2 RT-PCR test who had either been hospitalized because of COVID-19 or reported all of the following symptoms: fever > 38 °C, dyspnea, cough, and fatigue.
- *Mild COVID-19* Participants with a positive SARS-CoV-2 RT-PCR test not requiring hospitalization and who reported neither fever > 38 °C nor dyspnea.
- *Controls* This group consisted of both symptomatic and healthy controls. The symptomatic control group, designed similarly to the severe COVID-19 group, were those who had a negative SARS-CoV-2 RT-PCR test and who reported all of the following symptoms at the time of testing: fever > 38 °C, dyspnea, cough, and fatigue. The healthy control group reported none of the following symptoms during the three weeks preceding inclusion into the study: temperature > 38 °C, dyspnea, cough, fatigue, altered sense of smell and taste, sore throat, nasal symptoms, or headache, and no infections during the past six months. For this reason, the healthy controls had not undergone any SARS-CoV-2 RT-PCR testing.

Eligible participants from each group were randomly invited to donate a blood sample at the Oslo University Hospital. Of those who consented, a list of 318 randomly selected potential participants was prepared. The study staff called and invited participants into the substudy until approximately 200 appointments were made. To minimize sampling bias, all the participants were given the opportunity to choose their own time slot for the blood draw.

### Data collection

The evaluation of disease severity was based on self-reported information from the baseline questionnaire, which covered previous medical history, symptoms, disease duration, hospitalization, and remission. SARS-CoV-2 RT-PCR test results were obtained from the following four laboratories in the greater Oslo area: Fürst Medical laboratory, Oslo University Hospital, Akershus University Hospital, and Vestre Viken Hospital. A SARS-CoV-2 serology test was performed on all participants.

Evaluation of long-COVID was performed through an electronic follow-up questionnaire distributed three months after the blood draw. Reminders were sent to non-responders via email and SMS. Long-COVID was defined as a worsening in self-reported health from a year ago, assessed by a single-item, five-level question from the RAND 36-item health survey questionnaire^57^. This definition is considered to have high sensitivity for long-COVID. However, 12% of Norwegians reported a worsening in health in a general population survey based on this questionnaire^58^. Therefore, the specificity in the study population is estimated to be approximately 70%.

### Sampling scheme

Figure 1 provides a schematic overview of the sampling scheme used to assign the participants into distinct categories (i.e., severe COVID-19, mild COVID-19 and controls, and long-COVID and remission) according to the inclusion/exclusion criteria. Of the approximately 200 individuals who consented to a blood draw, 184 showed up for their appointment and completed the baseline questionnaire. Two of the participants failed the data file transfer, bringing the final number of participants included in the current analyses to 182. Of these, 48 were categorized as mild COVID-19, 61 as severe COVID-19, and 73 as controls. Based on the participants’ answers in the follow-up questionnaire three months after inclusion, we were able to reclassify four subjects from the mild and severe COVID-19 categories as belonging to the long-COVID category.

### Laboratory methods

The serum tubes were centrifuged, aliquoted, and frozen within four hours of the blood draw. The EDTA tubes with whole-blood samples were kept on ice, aliquoted, and frozen at − 80 °C within two hours of the blood draw. Confirmatory serology was performed based on the detection of anti-SARS-CoV-2 antibody against nucleocapsid, as measured by the Roche Cobas e601 module (Roche Diagnostics GmbH, Mannheim, Germany).

### DNAm measurement

DNA was extracted from 200 µl of EDTA-anticoagulated whole blood using the QIAsymphony DSP DNA Mini Kit (QIAGEN, catalog number 937236) at the Oslo University Hospital. The tubes were initially placed in random order but were not formally randomized before bisulfite conversion using the Zymo EZ-96DNA Methylation-Lightning MagPrep kit (Zymo Research, Irvine, USA). DNAm was measured using the Illumina Infinium MethylationEPIC BeadChip (Illumina, San Diego, USA) at Life & Brain GmbH, Bonn, Germany.

The raw signal intensity data were extracted from the IDAT files using a standard pipeline powered by the R packages minfi and DMRcate^59^. We applied the multiple sample/probe exclusion criteria prior to background correction and normalization. The detectionP function was used to exclude samples with a mean detection p-value greater than 0.01 and probes with a detection p-value greater than 0.01. Cross-reactive probes and probes within two base-pairs from a single-nucleotide polymorphism (SNP) with a minor allele frequency (MAF) greater than 0.05 were removed using the rmSNPandCH function in DMRcate. In addition, cross-hybridizing probes specific to the EPIC array, as previously reported by McCartney and co-workers^60^, were also excluded. In addition, the output of the minfi qcReport and plotQC were visually inspected for inconsistencies. Finally, the data were background corrected and normalized using the default settings of the preprocessFunnorm function in minfi.

Following the above QC steps, DNAm data on 182 individuals (109 individuals in the COVID-19 group and 73 in the non-COVID-19 group) and 794,075 probes remained for the current EWASs.

### Statistical analyses

#### EWAS

We stratified the methylation data into the following three subsets: (1) the combined sample of males and females and 776,892 autosomal probes, (2) males only and 17,183 sex chromosome probes (17,128 X-linked and 55 Y-linked), and (3) females only and 17,128 X-linked probes. In each subset, we compared the mean methylation levels, i.e., the M-values^61^, in the following group comparisons: (a) COVID-19 positive (n = 109) versus COVID-19 negative (n = 73), (b) severe COVID-19 (n = 61) versus mild COVID-19 (n = 48), and (c) long-COVID (n = 41) versus remission (n = 63). This analytic strategy is outlined in Fig. 2. The results from additional group comparisons can be found in Supplementary Figs. 8 and 9.

We fit linear regressions of the M-values on the COVID-19 variables, with adjustment for age, sex, imputed smoking pack-years, physical fitness, imputed white blood cell counts (CD8 + naïve and exhausted cytotoxic T cell, CD4 + naïve cells, natural killer cells, B cells, monocytes, and granulocytes), and plate. The imputed smoking pack-years and white blood cell counts were derived using Horvath's online calculator^62^. Next, we derived empirical Bayes moderated t-statistics and the corresponding P-values using the limma R package^63^.

The entire EWAS summary statistics can be found in Supplementary Files 1 and 2, respectively. All analyses were performed in the statistical programming language R, version 4.0.5.

#### Additional post-processing analyses

To identify differentially methylated regions (DMRs), we applied the dmrff function from the dmrff R package^64^ to the EWAS summary statistics. We chose dmrff because it was reported to be the most powerful method in a comparison involving four other popular methods for DMR detection (DMRcate, comb-p, seqlm, and GlobalP)^65^. The maximum distance between consecutive probes was set to 500 base-pairs (the default value). We defined a DMR as being statistically significant if it had a Bonferroni-corrected P-value less than 0.05.

Next, we performed a gene-set enrichment analysis of the significant CpGs detected by the EWASs to test for potential enrichment in biological pathways. We used the gometh function implemented in the missMethyl R package^66^, which queries Gene Ontology (GO) categories and Kyoto Encyclopedia of Genes and Genomes (KEGG) pathways.

We used the Illumina Infinium MethylationEPIC manifest file (v1.0 B5)^25^ to define and analyze the target genes. This file contains detailed information on whether a given CpG is located within specific regions of interest (e.g., gene-promoter region, promoter-flanking region, gene-body, CpG island, shelf, shore, and open sea), and whether the CpG is associated with specific regulatory features, such as DNase hypersensitive regions, chromatin regions, and enhancers (as defined by FANTOM5 annotations), etc.

Cell-type composition was estimated using the function estimateCellCounts in the minfi R package^67^.

#### Epigenetic age acceleration

Epigenetic age was estimated using the DNA methylation based age predictors DNAmAge^68^ and Hannum^69^ using the University of California Los Angeles (UCLA) web-based service^70^ that adjusts for blood cell composition. We used the (linear regression) residuals between estimated epigenetic age and true chronological age as the measurement of epigenetic age acceleration (EAA), as recommended by the authors of these epigenetic clocks. A positive EAA indicates that the epigenetic age is higher than the chronological age.

#### Ethics

The study was approved by the Regional Committees for Medical and Health Research Ethics (REK) in Norway (Reference Number 2021/8504) and conducted in accordance with the Declaration of Helsinki. All participants in the Norwegian Corona Cohort Study provided written informed consent.

## Supplementary Information

Supplementary Information 1.

Supplementary Information 2.

Supplementary Information 3.

Supplementary Information 4.

## Data Availability

To enhance data sharing and enable other researchers to compare their results with ours, we have provided the entire EWAS summary statistics for the joint analysis of males and females on 776,892 autosomal probes in Supplementary File 1. The corresponding EWAS summary statistics for the sex-stratified analyses of probes on the sex chromosomes (males and female separately on 17,183 sex-chromosome probes) are provided in Supplementary File 2. Due to written consent and ethical issues, the datasets in the current study are not publicly available. However, researchers may obtain a de-identified dataset upon reasonable request to the study authors and after approval from the study board. Data requests may be subjected to further review by the national register authority and the national ethics committee.
