# Supplementary material for: EWAS of post-COVID-19 patients shows methylation differences in the immune-response associated gene, IFI44L, three months after COVID-19 infection
Source: Sci Rep. 2022 Jul 7;12:11478. doi: 10.1038/s41598-022-15467-1 (PMC9261254; doi:10.1038/s41598-022-15467-1)
Supplement: Supplementary file 1 — Supplementary Information 1. [file 41598_2022_15467_MOESM1_ESM.pdf]

Supplementary materials to "*EWAS of post-COVID-19 patients shows methylation differences in the immune-response associated gene, IFI44L, three months after COVID-19 infection*"

Yunsung Lee[1,†], Espen Riskedal[2,†], Karl Trygve Kalleberg[2], Mette Istre[3], Andreas Lind[4], Fridtjof Lund-Johansen[5], Olaug Reiakvam[3], Arne V. L. Søraas[3], Jennifer R. Harris[1], John Arne Dahl[3], Cathrine L. Hadley[2,‡,*] and Astanand Jugessur[1,6,‡]

[1]Centre for Fertility and Health, Norwegian Institute of Public Health, P.O. box 222 Skøyen, 0213 Oslo, Norway

[2]Age Labs AS, Gaustadalléen 23A, 0373 Oslo, Norway

[3]Department of Microbiology, Oslo University Hospital Rikshospitalet, 0372, Oslo, Norway

[4]Department of Microbiology, Oslo University Hospital Ullevaal, 0450, Oslo, Norway

[5]Department of Immunology, Oslo University Hospital Rikshospitalet, 0372, Oslo, Norway

[6]Department of Global Public Health and Primary Care University of Bergen, P.O. box 7804, 5020 Bergen, Norway

† Joint first authors

‡ Joint senior authors

***Corresponding author:** Cathrine L. Hadley, MD PhD

Supplementary Figure 1. Quantile-Quantile plots for different comparisons in the pooled analyses of males and females

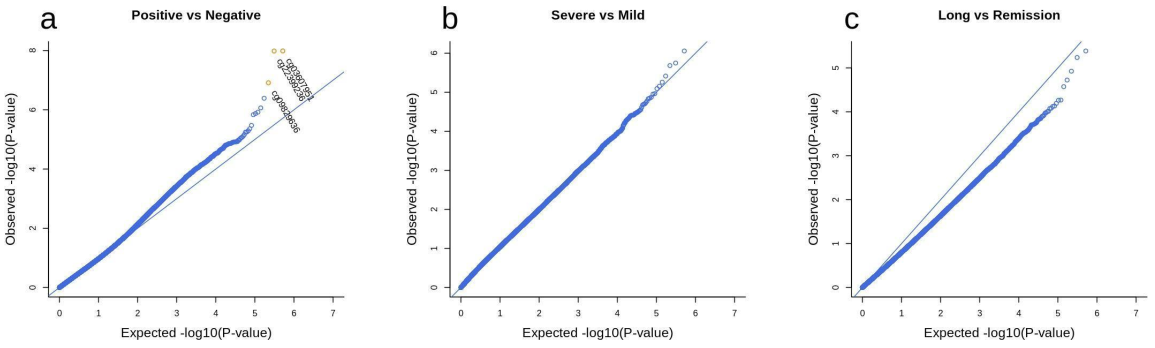

(a) COVID-19 positive (n=109) versus COVID-19 negative (n=73), inflation factor (lambda)=0.9677, **(b)** severe COVID-19 (n=61) versus mild COVID-19 (n=48), inflation factor (lambda)=1.0122, and **(c)** long-COVID (n=41) versus remission (n=63), inflation factor (lambda)=0.8974. The orange-colored dots in **(a)** are the significant CpG sites at FDR<0.05

Supplementary Figure 2. Quantile-Quantile plots for different comparisons in the sex-stratified analyses

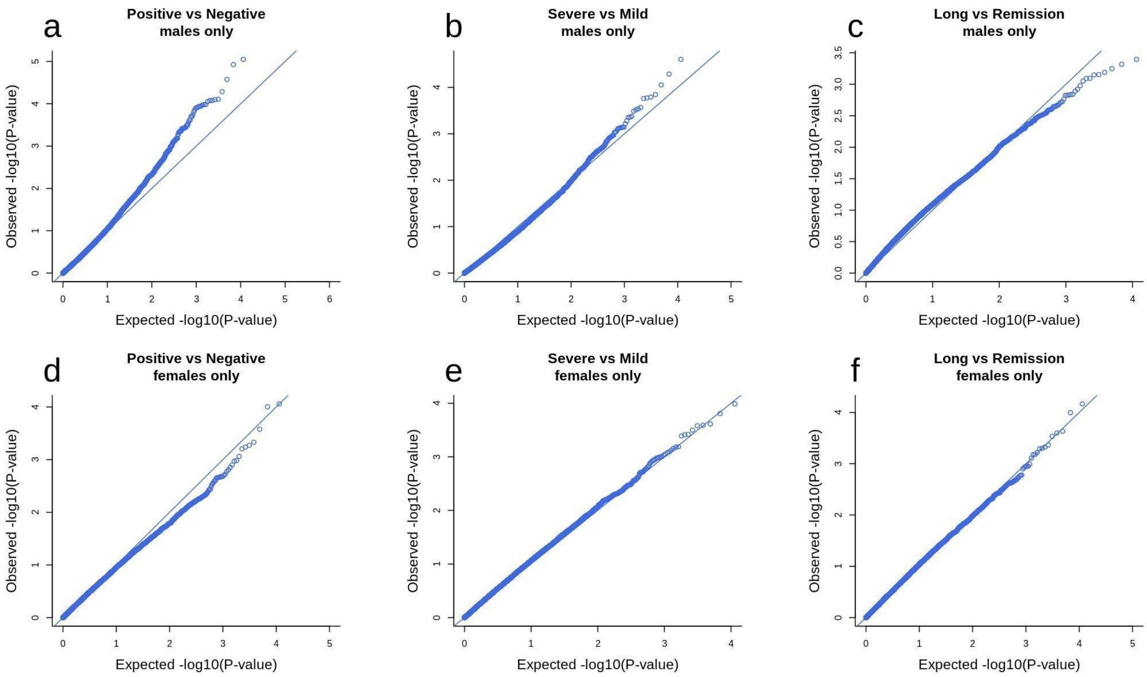

Panels **(a)** to **(c)** are for analyses in males and panels **(d)** to **(f)** are for analyses in females. Panels **(a)** and **(d)** show the comparison between COVID-19 positive (n=109) and COVID-19 negative (n=73) individuals. Panels **(b)** and **(e)** show the comparison between severe COVID-19 (n=61) and mild COVID-19 (n=48) individuals. Panels **(c)** and **(f)** show the comparison between individuals with long-COVID (n=41) and those in remission (n=63). The inflation factors (lambda values) are as follows: (a) lambda=1.0322, (b) lambda=0.9713, (c) lambda=1.0911, (d) lambda=0.9565, (e) lambda=1.0439, and (f) lambda=1.0282

Supplementary Figure 3. Comparison of the distribution of mean Beta-values for all X-linked probes in males and females

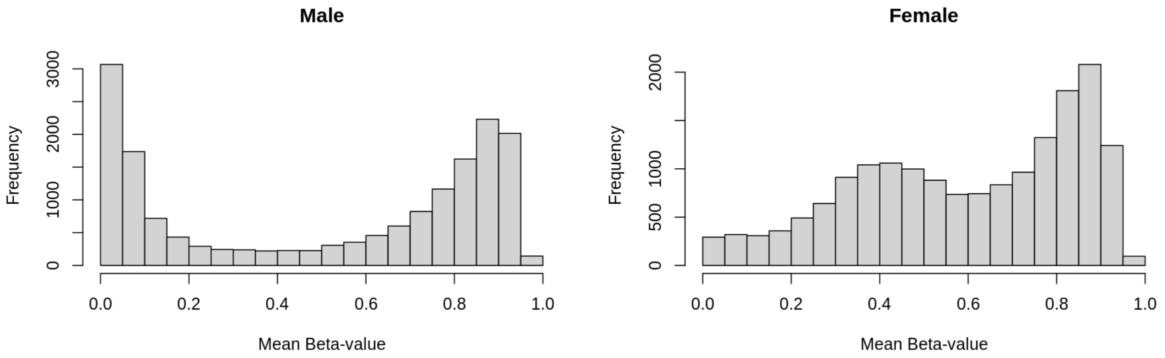

The characteristic hump in the middle of the distribution in females reflects X-chromosome inactivation (XCI). This is a process occurring in early embryonic development in which one of the X chromosomes in female somatic cells is randomly selected and transcriptionally inactivated, thus maintaining a similar dosage of X-linked genes in XX and XY individuals. The distinct sex difference in the distribution of mean Beta-values is the reason for conducting the current sex-stratified analyses of probes on the sex-chromosomes

Supplementary Figure 4. Boxplot of estimated cell-type compositions for the six different groups: Healthy controls, long-COVID, mild COVID-19, remission, severe COVID-19, and symptomatic controls

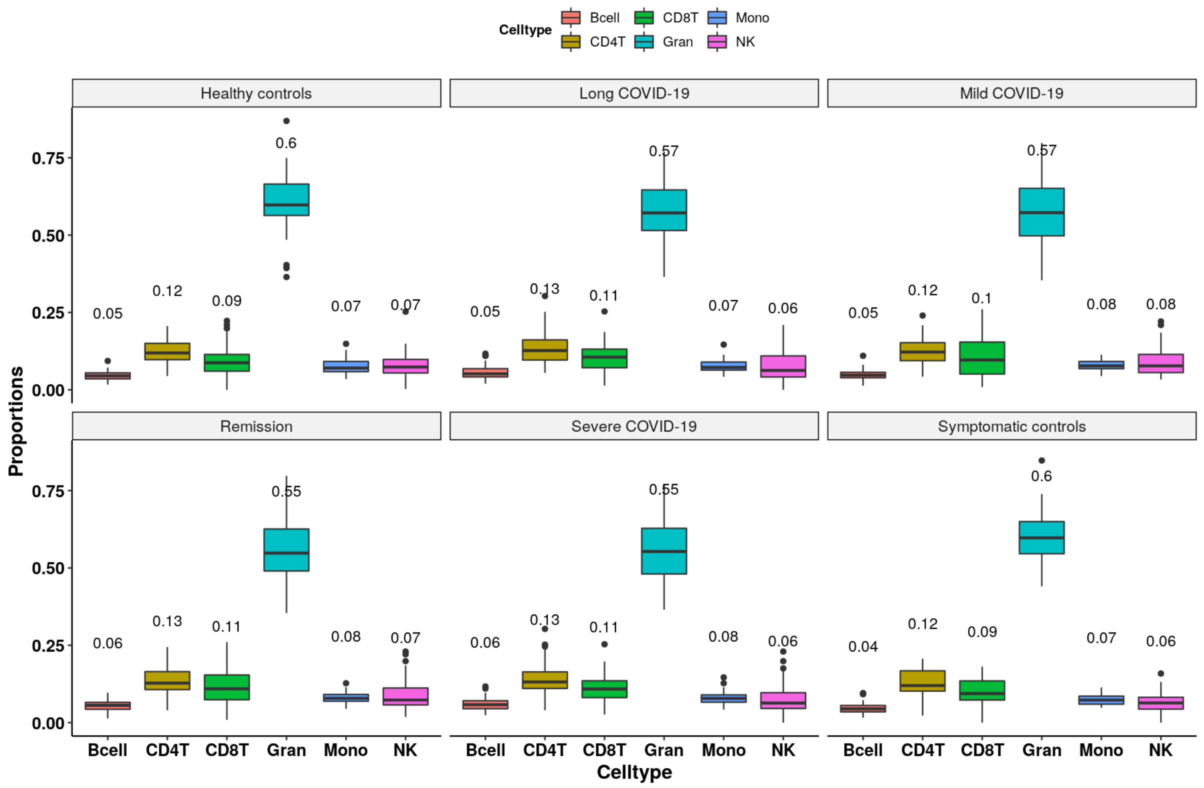

The plots display the estimated proportions of six main cell types in peripheral blood for each group (y-axis). The upper and lower box limits correspond to the interquartile range (25% to 75%) and the horizontal line in the box represents the median value. The median value for each cell type is also provided above the upper whisker. The whiskers outstretch 1.5 times the box height from the top and bottom of the box. The dots outside the whiskers represent outliers beyond the interquartile range. Abbreviations: Bcell: B-cell; CD4T: CD4+ T-cell; CD8T: CD8+ T-cell; Gran: granulocyte; Mono: monocyte; and NK: natural killer cell

Supplementary Figure 5. Boxplot of Wilcoxon rank sum tests comparing cell-type composition across the six groups: Healthy controls, long-COVID, mild COVID-19, remission, severe COVID-19, and symptomatic controls

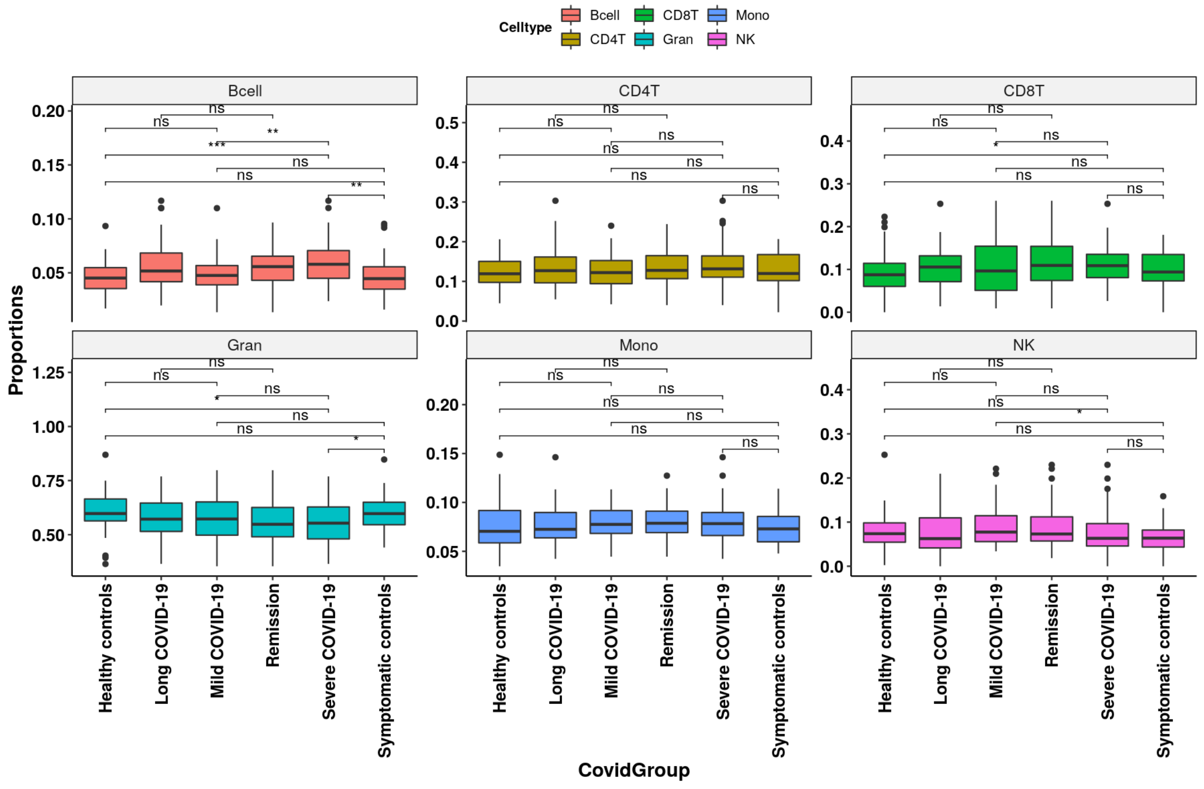

The plots display the estimated proportions of six main cell types in peripheral blood (y-axis). Each panel compares the proportions for one cell type across six groups using a Wilcoxon rank sum test. The following symbols indicate the level of statistical significance: ns=not significant, *=p≤ 0.05, **=p≤ 0.01 and ***=p ≤ 0.001. Abbreviations: Bcell: B-cell; CD4T: CD4+ T-cell; CD8T: CD8+ T-cell; Gran: granulocyte; Mono: monocyte; and NK: natural killer cell

Supplementary Figure 6. Comparison of epigenetic age acceleration between the groups, pooled analyses of males and females

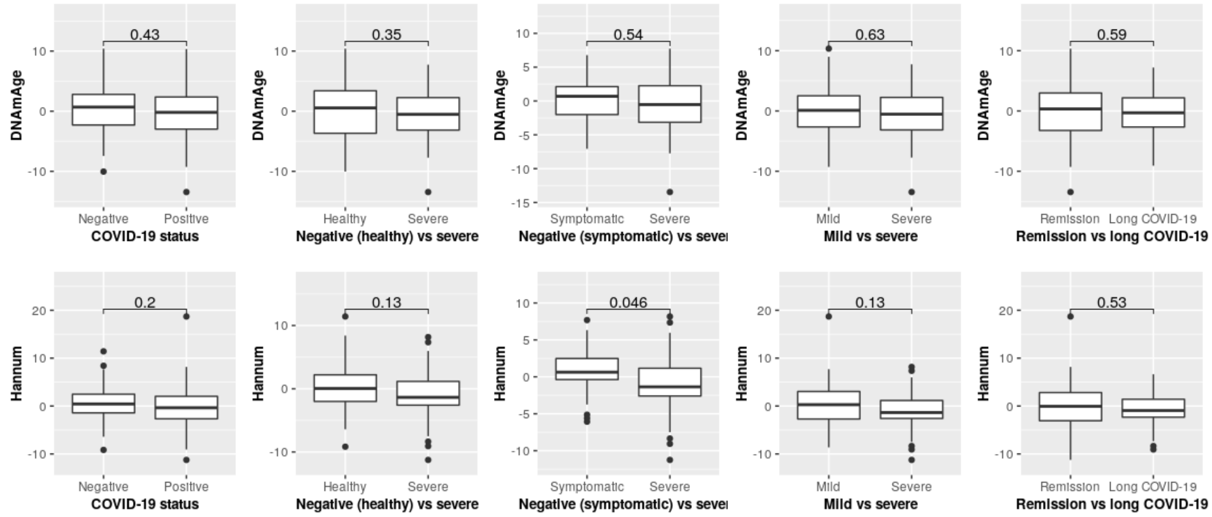

The box plots show the p-value of the mean difference in epigenetic age acceleration between the following pairs of groups: negative (all) versus positive (all), negative (healthy) versus positive (severe), negative (symptomatic) versus positive (severe), mild versus severe, long COVID versus remission. The first and second row compares age acceleration using the DNAmAge and Hannum epigenetic clocks, respectively. In each plot, the age acceleration residuals are used for comparison, as recommended by the authors of the epigenetic clocks. Only the Hannum clock exhibits a $p<0.05$ (negative symptomatic versus severe). P-values have not been corrected for multiple testing

Supplementary Figure 7. Groupwise comparison of differences in methylation of cg03607951 (*IFI44L*) in the following groups: severe versus mild COVID-19, long-COVID versus remission

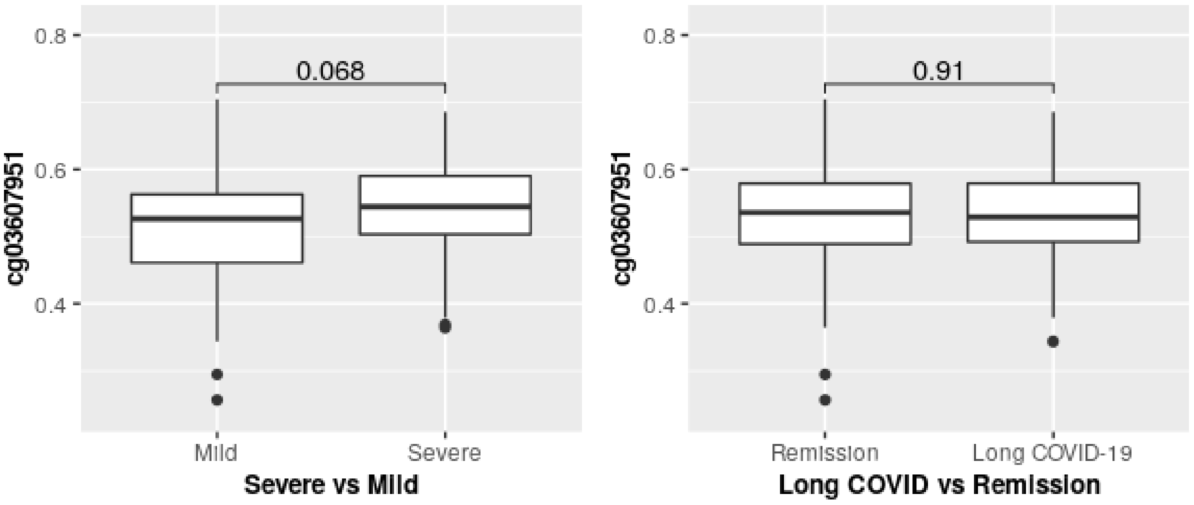

Note that the p-values have not been corrected for multiple testing

Supplementary Figure 8. Volcano plots for the pooled sample of males and females in the additional analysis of autosomal probes

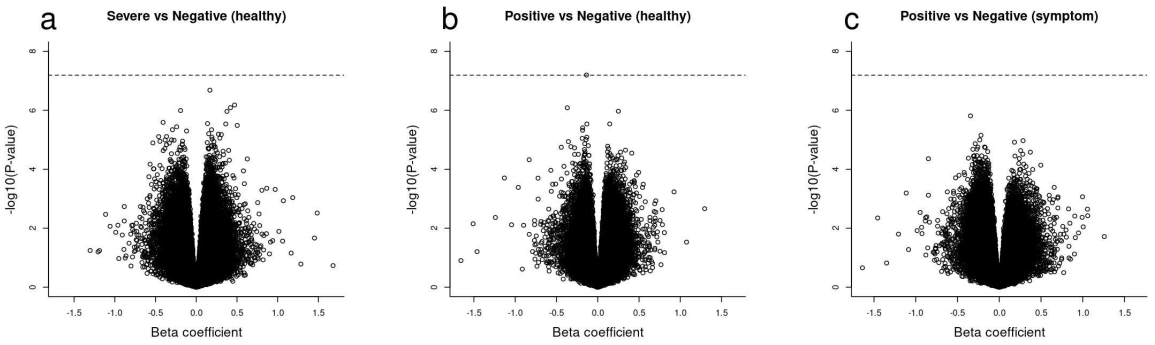

The plots display the estimated Beta coefficients against -$\log_{10}$ of the P-values. (a) severe COVID-19 (n=61) versus COVID-19 negative (n=41), (b) COVID-19 positive (n=109) versus healthy COVID-19 negative (n=41), and (c) COVID-19 positive (n=109) versus COVID-19 negative with symptoms (n=32). The dotted horizontal line refers to the Bonferroni threshold (0.05/776,892 CpG sites)

Supplementary Figure 9. Volcano plots for the additional sex-stratified analyses targeting sex-chromosome probes

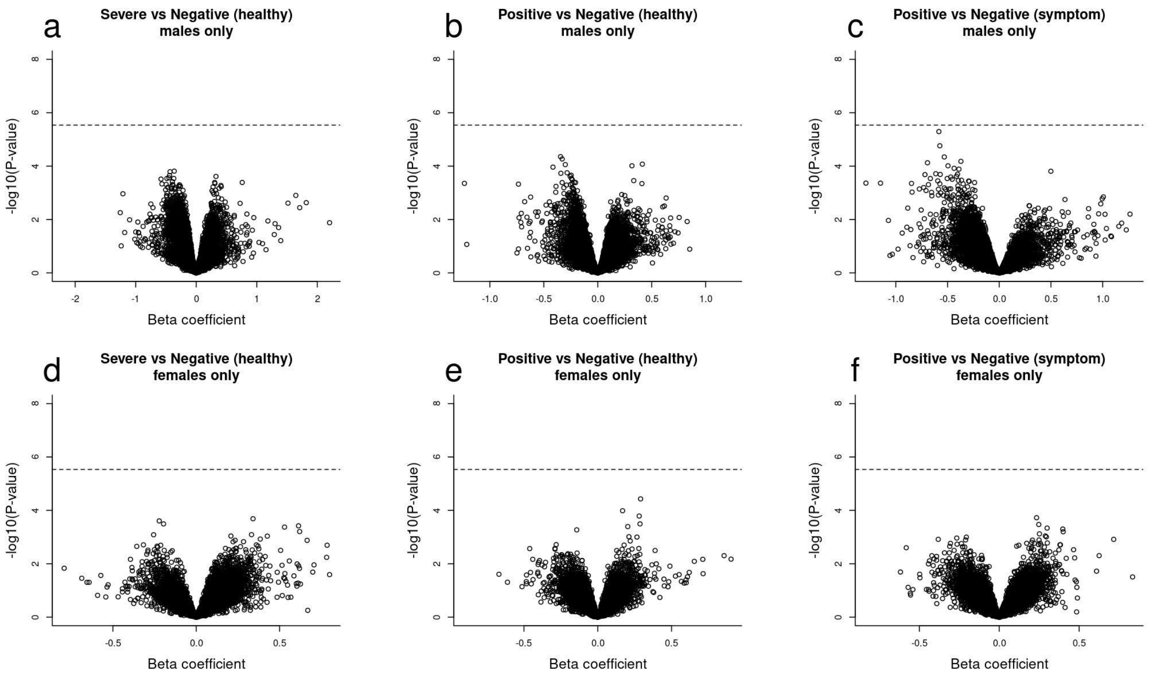

The plots display the estimated Beta coefficients against -$\log_{10}$ of the P-values. Panels (a) to (c) are for males and panels (d) to (f) are for females. Panels (a) and (d) show the results of the severe COVID-19 (n=61) versus healthy COVID-19 negative (n=41). Panels (b) and (e) show the results of the COVID-19 positive (n=109) versus healthy COVID-19 negative (n=41). Panels (c) and (f) show the results of the COVID-19 positive (n=109) versus COVID-19 negative with upper respiratory tract symptoms (n=32). The dotted horizontal line indicates the Bonferroni threshold (0.05/17, 183 CpGs for males and 0.05/17, 128 CpGs for females)

Supplementary Table 1. mQTLs associated with the three top CpGs detected in the EWAS of COVID-19 positive versus COVID-19 negative individuals

SNP	SNP Chr	SNP Position	A1	A2	MAF	CpG	CpG Chr	CpG Position	Beta	Effect Size	P-value
rs117341455	10	26355228	G	A	0.146	cg03607951	1	79085586	0.31221	0.03515	5.88e-08
rs4449117	2	62878660	C	T	0.329	cg03607951	1	79085586	0.22044	0.01095	9.55e-08
rs147755534	2	62879422	T	C	0.329	cg03607951	1	79085586	0.22044	0.01095	9.55e-08
rs2421713	2	62878720	G	A	0.329	cg03607951	1	79085586	0.22044	0.01095	9.55e-08
rs11532579	2	62878360	G	T	0.329	cg03607951	1	79085586	0.22036	0.01180	8.93e-08
rs6731286	2	62839819	A	T	0.336	cg03607951	1	79085586	0.21880	0.01141	7.68e-08
rs6734002	2	62873916	T	G	0.335	cg03607951	1	79085586	0.22267	0.01117	8.10e-08
rs6741300	2	62877346	A	G	0.34	cg03607951	1	79085586	0.22815	0.01221	3.07e-08
rs9678363	2	62879168	G	A	0.329	cg03607951	1	79085586	0.22044	0.01095	9.55e-08
rs2901517	2	62878753	C	T	0.329	cg03607951	1	79085586	0.22044	0.01095	9.55e-08
rs13403509	2	62878163	G	A	0.33	cg03607951	1	79085586	0.22573	0.01106	4.74e-08
rs6713090	2	62877386	G	A	0.342	cg03607951	1	79085586	0.23400	0.01221	1.42e-08
rs11055131	12	13041772	G	C	0.043	cg09829636	14	102976856	0.57985	0.00502	6.10e-08
rs10845663	12	13041562	T	G	0.042	cg09829636	14	102976856	0.58219	0.00501	6.50e-08
rs11055129	12	13040563	A	G	0.042	cg09829636	14	102976856	0.58219	0.00501	6.50e-08
rs56295645	12	13041091	G	C	0.042	cg09829636	14	102976856	0.58219	0.00501	6.50e-08
rs61912337	12	13045949	G	A	0.046	cg09829636	14	102976856	0.58538	0.00503	2.57e-08

Supplementary File 1. EWAS summary statistics for the pooled analysis of males and females performed on 776,892 autosomal probes

Supplementary File 2. EWAS summary statistics for the sex-stratified analysis performed on 17,183 sex-chromosome probes

Supplementary File 3. Differentially methylated regions (DMRs) identified in the different group comparisons
